# A clinical risk score to predict 3-, 5- and 10-year survival in patients undergoing surgery for Dukes B colorectal cancer

**DOI:** 10.1038/sj.bjc.6605864

**Published:** 2010-08-31

**Authors:** D C McMillan, C S McArdle, D S Morrison

**Affiliations:** 1 University Department of Surgery, Faculty of Medicine – University of Glasgow, Royal Infirmary, Glasgow G31 2ER, UK; 2 West of Scotland Cancer Surveillance Unit, Section of Public Health and Health Policy, Faculty of Medicine – University of Glasgow, G12 8RZ, UK

**Keywords:** colorectal cancer, age, mode of presentation, curative surgery, anastomotic leakage, survival

## Abstract

**Background:**

The prognosis of patients with Dukes stage B colorectal cancer is unpredictable and there is continuing interest in simply and reliably identifying patients at high risk of developing recurrence and dying of their disease. The aim of this study was to devise a clinical risk score to predict 3-, 5- and 10-year survival in patients undergoing surgery for Dukes stage B colorectal cancer.

**Methods:**

A total of 1350 patients who underwent surgery for Dukes stage B colorectal cancer between 1991 and 1994 in 11 hospitals in Scotland were included in the analysis.

**Results:**

On follow-up, 926 patients died of whom 479 died of their cancer. At 10 years, cancer-specific survival was 61% and overall survival was 38%. On multivariate analysis, age ⩾75 (hazard ratio (HR) 1.45, 95% confidence interval (CI) 1.15–1.82, *P*=0.001), emergency presentation (HR 1.59, 95% CI 1.27–1.99, *P*<0.001) and anastomotic leak (HR 2.17, 95% CI 1.24–3.78, *P*<0.01) were independently associated with cancer-specific survival in colon cancer. On multivariate analysis, only age ⩾75 (HR 1.58, 95% CI 1.14–2.18, *P*<0.01) was associated with cancer-specific survival in rectal cancer. Age, presentation and anastomotic leak hazards could be simply added to form a clinical risk score from 0 to 2 in colon cancer. In patients with Dukes B stage colon cancer, the cancer-specific survival at 5 years for patients with a cumulative score 0 was 81%, 1 was 67% and 2 was 63%. The cancer-specific survival rate at 10 years for patients with a clinical risk score of 0 was 72%, 1 was 58% and 2 was 53%.

**Conclusion:**

The results of this study, in a mature cohort, introduce a new simple clinical risk score for patients undergoing surgery for Dukes B colon cancer. This provides a solid foundation for the examination of the impact of additional factors and treatment on prediction of 3-, 5- and 10-year cancer-specific survival.

Colorectal cancer is the second commonest cause of cancer death in Western Europe and North America (Parkin *et al*, 2005). Many patients have evidence of locally advanced or metastatic disease at the time of initial presentation. Even in those undergoing apparently curative resection for Dukes stage B disease, approximately one-third will die of their disease within 5 years (McArdle and Hole, 2002; Morris *et al*, 2006). In view of these poor results, there is increasing interest in the use of adjuvant chemotherapy in these patients.

The treatment for Dukes B colorectal cancer remains primarily based on surgery alone and adjuvant chemotherapy is not routinely given. Conventionally, in these patients the decision whether or not to offer adjuvant 5-fluorouracil-based chemotherapy is mainly based on the patient's age and fitness to tolerate chemotherapy. However, even in this selected cohort, the impact of chemotherapy on outcome is unpredictable. Therefore, there is continuing interest in simply and reliably identifying patients at high risk of developing recurrence and dying of their disease (Cascinu *et al*, 2003; Benson *et al*, 2004). The significance of this problem is increasing with the widespread introduction of screening programmes and the consequent increase in proportion of patients presenting with early-stage disease (Benson, 2007).

On the basis of analysis of large data sets, there is reliable information that a number of routinely collected factors influence outcome following surgery for Dukes B colorectal cancer. These include older age (Mulcahy *et al*, 1994; Shankar and Taylor, 1998; McMillan *et al*, 2008), male gender (McArdle *et al*, 2003; Paulson *et al*, 2009), socioeconomic deprivation (Hole and McArdle, 2002; Kelsall *et al*, 2009), tumour site (McArdle and Hole, 2002), emergency presentation (McArdle and Hole, 2004a; Wong *et al*, 2008), surgeon specialisation (McArdle and Hole, 2004b; Renzulli *et al*, 2006) and anastomotic leakage (McArdle *et al*, 2005; Law *et al*, 2007).

Therefore, the aim of this study was to devise a clinical risk score to predict 3-, 5- and 10-year survival in patients undergoing surgery for Dukes stage B colorectal cancer.

## Patients and methods

A total of 1411 patients who underwent a resection for Dukes B colorectal cancer between 1 January 1991 and 31 December 1994 in 11 hospitals in the central belt of Scotland were included in the study. Information was abstracted from case notes for different patients by two specially trained data managers. Details included age, sex, deprivation category (DEPCAT), site of tumour, Dukes’ stage, the nature of surgery, postoperative mortality and adjuvant therapy. Data for 1991 and 1992 were collected retrospectively, and those for 1993 and 1994 were collected prospectively. There was no difference in baseline characteristics of the patients between the two periods.

Cancer-specific deaths were determined as a first, or principal underlying cause of death with International Classification of Diseases (ICD) 9 codes for colon and rectal cancers, 153 and 154, as well as 150, 157, 159, 199 and by ICD-10 codes C18 to 20, as well as C15, C25, C26 and C80. Overall survival was determined as deaths from any cause. Death records were complete until 28 September 2007 and this was therefore the censor date for all individuals who had not died.

Patients’ socioeconomic circumstances were inferred using the DEPCAT, a validated categorical score that ranks residential postcodes from 1 (most affluent) to 7 (most deprived) using four Census variables that were found to best predict health outcomes – car ownership, unemployment, overcrowding and lower occupational social classes (IV and V) (Carstairs and Morris, 1991). They were further grouped into three conventional categories: 1 and 2 (affluent); 3–5 (intermediate); and 6 and 7 (deprived).

Patients who were considered as an emergency had either presented with significant blood loss, obstruction or perforation (McArdle *et al*, 2006). Tumours were classified according to site, colon or rectum. The extent of tumour spread was assessed by conventional Dukes’ classification based on histological examination of the resected specimen. Patients were deemed to have had a curative resection if the surgeon considered that there was no macroscopic residual tumour once resection had been completed. Individual surgeons were defined as specialists or non-specialists by a panel of six senior consultants and one of the authors (CSMcA). These assessments were made without the knowledge of the outcome and before any analysis was performed.

Approval was obtained for information on date and cause of death to be checked with that received by the cancer registration system through linkage with the Registrar General (Scotland). Deaths up to 28 September 2007 have been included in the analysis, providing a median follow-up time of 14.6 years (minimum 13 years, maximum 17 years).

### Statistical analysis

The grouping of variables was carried out using conventional categories. Univariate and multivariate survival analysis and calculation of hazard ratios (HRs) were carried out using Cox's proportional hazards model. The proportionality assumption was tested by visual inspection of log-minus-log plots. Interactions between variables in the multivariate analyses were tested by the addition of all possible pairwise interaction terms. Cumulative survival following colorectal cancer surgery was estimated using the Kaplan–Meier method and the log-rank used to test for independence between variables. Predictive model analysis using receiver operating characteristic analysis was carried out. C-statistics were calculated with the null hypothesis that the true area under the curve was 0.5, and asymptotic 95% confidence intervals (CIs) calculated around the best estimate. Analysis was performed using the SPSS software package version 15.0 (SPSS Inc., Chicago, IL, USA).

## Results

Of the 1411 patients who underwent a resection for Dukes B colorectal cancer, there were 61 postoperative deaths and 1350 patients were included in the analysis. The majority were aged <75 years (64%), were not socioeconomically deprived (80%), presented electively (71%), had colonic tumours (68%) and were treated by a general surgeon (74%). A total of 45 (3%) patients developed an anastomotic leak and 36 (3%) patients received adjuvant therapy. On follow-up, 926 patients died of whom 479 died of their cancer. At 10 years, cancer-specific survival was 61% and overall survival was 38%, giving median survival times of 10.0 and 6.3 years, respectively.

The relationship between clinicopathological characteristics and cancer-specific survival in patients with colon cancer is shown in Table 1. On univariate analysis, age (*P*<0.01), mode of presentation (*P*<0.001) and anastomotic leak (*P*<0.01) were significantly associated with cancer-specific survival. On multivariate analysis of these significant factors, age ⩾75 (HR 1.45, 95% CI 1.15–1.82, *P*=0.001), emergency presentation (HR 1.59, 95% CI 1.27–1.99, *P*<0.001) and anastomotic leak (HR 2.17, 95% CI 1.24–3.78, *P*<0.01) were independently associated with cancer-specific survival (Table 1). There were no significant interactions between any combination of age, presentation and anastomotic leak in the colon model.

The relationship between clinicopathological characteristics and cancer-specific survival in patients with rectal cancer is shown in Table 2. On univariate analysis, age (*P*<0.01) and mode of presentation (*P*<0.05) were significantly associated with cancer-specific survival. On multivariate analysis of these significant factors, only age ⩾75 (HR 1.58, 95% CI 1.14–2.18, *P*<0.01) was independently associated with cancer-specific survival (Table 2).

The relationship between clinicopathological characteristics and overall survival in patients with colon cancer is shown in Table 3. On univariate analysis, age (*P*<0.001), sex (*P*<0.01) and mode of presentation (*P*<0.001) were significantly associated with overall survival. On multivariate analysis of these significant factors, age ⩾75 (HR 2.15, 95% CI 1.84–2.52, *P*<0.001), sex (HR 1.30, 95% CI 1.11–1.52, *P*=0.001) and emergency presentation (HR 1.44, 95% CI 1.22–1.69, *P*<0.001) were independently associated with overall survival (Table 3). There were no significant interactions between any combination of age, presentation and anastomotic leak in the colon model.

The relationship between clinicopathological characteristics and overall survival in patients with rectal cancer is shown in Table 4. On univariate analysis, age (*P*<0.001) and mode of presentation (*P*<0.10) were significantly associated with overall survival. However, on multivariate analysis of these significant factors, only age ⩾75 (HR 2.20, 95% CI 1.74–2.79, *P*<0.001) was independently associated with overall survival (Table 4).

**Figure 1:**
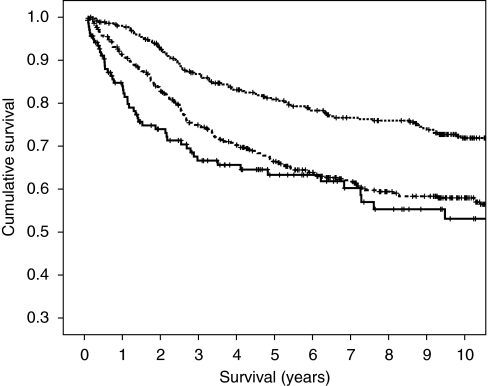
The relationship between a clinical risk score (0, 1 and 2 from top to bottom) and cancer-specific survival in patients undergoing surgery for Dukes B colon cancer.

With reference to cancer-specific survival in colon cancer, as the magnitude of the covariates of age ⩾75 (1.45), emergency presentation (1.59) and anastomotic leak (2.17) were similar, they could be allocated a score of 1 if they occurred or 0 if absent. Together these factors could be simply added to form a clinical risk score from 0 to 3. From the Kaplan–Meier curve of this clinical risk score it appeared that there was overlap in cancer-specific survival between clinical risk scores 2 and 3 and therefore these were combined to give clinical risk scores of 0, 1 and 2. The relationship between such a cumulative prognostic score and 3-, 5- and 10-year cancer-specific survival is shown in Figure 1.

In patients with Dukes B stage colon cancer, the cancer-specific survival rates at 3 years for patients with a cumulative score 0 was 87%, 1 was 75% and 2 was 67% (Table 5). The cancer-specific survival rates at 5 years for patients with a cumulative score 0 was 81%, 1 was 67% and 2 was 63%. The cancer-specific survival rates at 10 years for patients with a cumulative score 0 was 72%, 1 was 58% and 2 was 53%. The area under the curve for the clinical risk score with cancer mortality as an end point at 3, 5 and 10 years was (0.603, 95% CI 0.559–0.648, *P*<0.001), (0.582, 95% CI 0.541–0.623, *P*<0.001) and (0.561, 95% CI 0.522–0.600, *P*=0.003), respectively.

## Discussion

The results of this study show that in a large mature cohort of patients undergoing resection for Dukes B colon and rectal cancer, there were a number of clinical factors that were associated with poorer cancer-specific survival. In colon cancer age, mode of presentation and anastomotic leak were independently associated with cancer-specific survival. In rectal cancer, only advanced age was independently associated with cancer-specific survival. The reliable identification of these factors enabled the formation of a simple clinical risk score for colon cancer that clearly identified differences in 3-, 5- and 10-year cancer-specific survival.

It was of interest that this simple clinical risk score identified variations in 5-year cancer-specific survival of between 81 and 63% in patients with Dukes B colon cancer. These results have a number of important implications. First, simple nomograms from the present paper can help clinicians to readily identify those patients at higher risk of developing recurrence and dying of their disease. Second, these scores provide simple stratification factors for clinical studies and trials. Third, the score may provide a basis for future staging systems for Dukes B colorectal cancer to which further discriminatory variables might be added.

In this study, because of its potential impact on clinical practice, the main focus was on the factors that were independently associated with cancer-specific survival. However, it was of interest that, on 10-year follow-up, almost as many patients died of intercurrent disease (*n*=447) as died of their cancer (*n*=479). Given that many registries in different countries report the date of death, but not the cause of death, it was of interest that the significant independent factors in this study, age and mode of presentation, were similarly associated with overall survival.

In this study, a number of more recently recognised tumour prognostic factors such as intra or extramural vascular invasion, peritoneal involvement, margin involvement and tumour perforation (Roxburgh *et al*, 2009) were not available for analysis. Also, recent web-based prognostic calculators developed to individualise decisions regarding adjuvant therapy in patients with pathological TNM stage II and III colon cancer (Bardia *et al*, 2010), have included T stage and tumour grade (Numeracy, www.mayoclinic.com/calcs) and comorbidity and the number of examined lymph nodes (Adjuvant!, www.adjuvantonline.com). These were not available in the present analysis. Since very few patients in this study received either adjuvant (<3%) or neoadjuvant therapy (0%), the effect of therapy in colon and rectal cancer could not be examined. Furthermore, new approaches to staging the host inflammatory response, such as the Glasgow Prognostic Score (McMillan, 2009; Roxburgh and McMillan, 2010) were not available in the present analysis. Nevertheless, the present clinical risk score in a mature cohort provides a solid foundation for the examination of the impact of these additional factors and treatment on prediction of 3-, 5- and 10-year cancer-specific survival in patients undergoing surgery for Dukes B colon cancer.

It may be hypothesised that the effects of age, mode of presentation and anastomotic leak on cancer-specific survival are biologically mediated and therefore likely to be generalisable to other tumour types. In this study, mode of presentation and anastomotic leak were not significantly independently associated with cancer-specific survival in rectal cancer. However, the number of rectal cancers was less than half that of the colon cancers and less than 500. It is of interest that a number of recent reports, in larger cohorts, indicate that postoperative anastomotic leakage (Ptok *et al*, 2007; Sierzega *et al*, 2010) is also associated with poor long-term survival, independent of tumour staging, in rectal and gastric cancer. Therefore, aspects of the present simple clinical risk score, for patients with Dukes stage B colon cancer, may be useful in patients undergoing surgery for other gastrointestinal cancers.

In summary, the results of this study, in a mature cohort, introduce a new simple clinical risk score for patients undergoing surgery for Dukes B colon cancer. This provides a solid foundation for the examination of the impact of additional factors and treatment on prediction of 3-, 5- and 10-year cancer-specific survival.

## Figures and Tables

**Table 1:** The relationship between clinicopathological characteristics and cancer-specific survival in patients undergoing surgery for Dukes B colon cancer: univariate and multivariate analysis

		**Univariate analysis**		**Multivariate analysis**	
**Risk factor**	**Patients (*n*=920)**	**Hazard ratio (95% CI)**	***P*-value**	**Hazard ratio (95% CI)**	***P*-value**
Age (<75/⩾75 years)	562/358	1.43 (1.14–1.79)	0.002	1.45 (1.15–1.82)	0.001
Sex (female/male)	493/427	1.22 (0.98–1.53)	0.081		
Deprivation^a^ (intermediate)	164/589/166	1.15 (0.85–1.57)	0.369		
(deprived)		1.06 (0.72–1.56)	0.780		
Mode of presentation (elective/emergency)	592/328	1.58 (1.26–1.98)	<0.001	1.59 (1.27–1.99)	<0.001
Specialisation (yes/no)	197/700	1.19 (0.89–1.59)	0.241		
Anastomotic leak (no/yes)	896/24	2.11 (1.21–3.67)	0.009	2.17 (1.24–3.78)	0.006
Adjuvant therapy (no/yes)	811/14	0.76 (0.28–2.03)	0.577		

Abbreviation: CI=confidence interval.

a Baseline – affluent.

Baseline variables/comparison group in brackets after risk factor.

**Table 2:** The relationship between clinicopathological characteristics and cancer-specific survival in patients undergoing surgery for Dukes B rectal cancer: univariate and multivariate analysis

		**Univariate analysis**		**Multivariate analysis**	
**Risk factor**	**Patients (*n*=430)**	**Hazard ratio (95% CI)**	***P*-value**	**Hazard ratio (95% CI)**	***P*-value**
Age (<75/⩾75 years)	302/128	1.65 (1.19–2.27)	0.002	1.58 (1.14–2.18)	0.006
Sex (female/male)	186/244	1.18 (0.87–1.60)	0.298		
Deprivation^a^ (intermediate)	65/260/105	0.95 (0.63–1.45)	0.812		
(deprived)		0.93 (0.58–1.50)	0.767		
Mode of presentation (elective/emergency)	364/66	1.56 (1.06–2.28)	0.023	1.44 (0.98–2.12)	0.062
Specialisation (yes/no)	122/299	1.09 (0.77–1.53)	0.625		
Anastomotic leak (no/yes)	409/21	1.25 (0.66–2.37)	0.495		
Adjuvant therapy (no/yes)	348/22	1.62 (0.88–3.01)	0.124		

Abbreviation: CI=confidence interval.

a Baseline – affluent.

Baseline variables/comparison group in brackets after risk factor.

**Table 3:** The relationship between clinicopathological characteristics and overall survival in patients undergoing surgery for Dukes B colon cancer: univariate and multivariate analysis

		**Univariate analysis**		**Multivariate analysis**	
	**Patients (*n*=920)**	**Hazard ratio (95% CI)**	***P*-value**	**Hazard ratio (95% CI)**	***P*-value**
Age (<75/⩾75 years)	562/358	2.13 (1.81–2.49)	<0.001	2.15 (1.84–2.52)	<0.001
Sex (female/male)	493/427	1.26 (1.07–1.47)	0.005	1.30 (1.11–1.52)	0.001
Deprivation^a^ (intermediate)	164/589/166	1.22 (0.97–1.52)	0.089		
(deprived)		1.28 (0.98–1.67)	0.076		
Mode of presentation (elective/emergency)	592/328	1.41 (1.20–1.65)	<0.001	1.44 (1.22–1.69)	<0.001
Specialisation (yes/no)	197/700	0.92 (0.76–1.11)	0.362		
Anastomotic leak (no/yes)	896/24	1.56 (0.99–2.46)	0.057		
Adjuvant therapy (no/yes)	811/14	0.48 (0.22–1.08)	0.077		

Abbreviation: CI=confidence interval.

a Baseline – affluent.

Baseline variables/comparison group in brackets after risk factor.

**Table 4:** The relationship between clinicopathological characteristics and overall survival in patients undergoing surgery for Dukes B rectal cancer: univariate and multivariate analysis

		**Univariate analysis**		**Multivariate analysis**	
	**Patients (*n*=430)**	**Hazard ratio (95% CI)**	***P*-value**	**Hazard ratio (95% CI)**	***P*-value**
Age (<75/⩾75 years)	302/128	2.26 (1.78–2.85)	<0.001	2.20 (1.74–2.79)	<0.001
Sex (female/male)	186/244	1.12 (0.89–1.41)	0.324		
Deprivation^a^ (intermediate)	65/260/105	1.18 (0.84–1.64)	0.345		
(deprived)		1.22 (0.84–1.77)	0.300		
Mode of presentation (elective/emergency)	364/66	1.47 (1.09–1.97)	0.011	1.30 (0.97–1.75)	0.084
Specialisation (yes/no)	122/299	1.04 (0.80–1.34)	0.779		
Anastomotic leak (no/yes)	409/21	1.08 (0.64–1.81)	0.777		
Adjuvant therapy (no/yes)	348/22	1.16 (0.69–1.95)	0.589		

Abbreviation: CI=confidence interval.

a Baseline – affluent.

Baseline variables/comparison group in brackets after risk factor.

**Table 5:** The relationship between a clinical risk score and cancer-specific survival in patients undergoing surgery for colon cancer

	**Dukes B**	**Hazard ratio**		**Cancer-specific survival rate, % (SE)**		
	***n*=920 (%)**	**(95% CI)**	***P*-value**	**3 year**	**5 year**	**10 year**
Clinical risk score 0	355 (39)	1		87 (2)	81 (2)	72 (3)
Clinical risk score 1	425 (46)	1.80 (1.39–2.32)	<0.001	75 (2)	67 (2)	58 (3)
Clinical risk score 2	140 (15)	2.31 (1.64–3.26)	<0.001	67 (4)	63 (5)	53 (5)

Abbreviations: CI=confidence interval; SE=standard error.
